# Termination of Repeat Testing in Chemical Laboratories Based on Practice Guidelines: Examining the Effect of Rule-Based Repeat Testing in a Transplantation Center

**DOI:** 10.1155/2021/9955990

**Published:** 2021-05-13

**Authors:** Neda Soleimani, Amir Azadi, Mohammad Javad Esmaeili, Fatemeh Ghodsi, Reza Ghahramani, Azadeh Hafezi, Tayebeh Hosseyni, Arezoo Arabzadeh, Samira khajeh, Mahsa Farhadi, Sahand Mohammadzadeh

**Affiliations:** ^1^Department of Pathology, Shiraz Medical School, Shiraz University of Medical Sciences, Shiraz, Iran; ^2^Department of Pathology, Shiraz Transplant Center, Abu Ali Sina Hospital, Shiraz University of Medical Sciences, Shiraz, Iran

## Abstract

**Background:**

Although the automation of instruments has reduced the variability of results and errors of analysis, in some laboratories, repeating a test to confirm its accuracy is still performed for critical and noncritical results. However, the importance of repeat testing is not well established yet, and there are no clear criteria for repeating a test.

**Materials and Methods:**

In this cross-sectional study, all repeated tests for 26 biochemical analytes (i.e., albumin, alkaline phosphatase (ALP), alanine aminotransferase (ALT), amylase, aspartate aminotransferase (AST), bilirubin total (BT), bilirubin direct (BD), blood urea nitrogen (BUN), calcium, chloride (Cl), cholesterol total (CholT), creatine kinase (CK), creatinine (Cr), glucose, gamma-glutamyl transferase (GGT), high-density lipoprotein-cholesterol (HDL-c), iron, lactate dehydrogenase (LDH), LDL-c, lipase, magnesium (Mg), phosphorus (Ph), protein total (ProtT), total iron binding capacity (TIBC), triglyceride (TG), and uric acid) were assessed in both critical and noncritical ranges over two consecutive months (routine subjective test repeats in the first month and rule-based repeats in the second month). To determine the usefulness of test repeats, differences between the initial and verified results were compared with the allowable bias, and repeat testing was considered necessary if it exceeded the allowable bias range. All causes of repeat testing, including linearity flags, delta checks, clinically significant values, and critical values, were also documented. All data, including the cause of repeats, initial and verified results, time, and costs in the two consecutive months, were transferred to Microsoft Excel for analysis. For comparison of data between the months, Student's *t*-test was used.

**Results:**

A total of 7714 repeat tests were performed over two consecutive months. Although a significant decline (38%) was found in repeated tests in the second month (*P* < 0.001), there was no significant change in the percentage of unnecessary repeats (77% in the first month and 74% in the second month). In both consecutive months, AST and ALT were the most commonly repeated tests, and delta check was the most common cause of repeat testing. Mg, ALP, AST, and lipase showed the highest rates of necessary repeats, respectively (the least stable tests), while albumin, LDL, and CholT tests showed the highest rates of unnecessary repeats, respectively (the most stable tests). The total cost and delay in turnaround time (TAT) due to repeated testing decreased by 32% and 36%, respectively.

**Conclusion:**

Although repeat testing has been shown to be unnecessary in most cases, having a strict policy for repeat testing appears to be more valuable than avoiding it completely. Each laboratory is advised to establish its own protocol for repeat testing based on its own practice.

## 1. Introduction

The delivery of proficient laboratory services is the cornerstone of modern healthcare systems, contributing to about 70% of all medical decisions [1]. Providing high-quality and uninterrupted laboratory services requires many procedures to prevent problems. With the current technological advances, the accuracy of analyses has increased significantly, making today's equipment much more accurate than before. In contrast to preanalysis and postanalysis stages (as the main sources of laboratory error), the analysis phase accounts for about 0.1–3.8% of total laboratory errors [2–5].

So far, repeat testing has been a component of laboratory quality control activities for verification of data prior to releasing them out. Although in the analysis stage, automation of instruments has reduced the variability of results and errors, in some laboratories, repeating a test to ensure its accuracy is still performed for critical and also noncritical results [2, 6]. The main causes of repeat testing include values above and below the analytical measurement range (AMR), delta checks, clinically significant values, and critical values. A large number of repeated data reported by clinical biochemistry laboratories agree with the initial findings and are classified as unnecessary repeats when evaluated based on the criteria.

Unfortunately, there are no definite criteria to repeat a test. The test repetitions, which are not based on any specific criteria, not only cause an increase in the turnaround time (TAT) or delayed reporting but also consume reagents that consequently increase the cost of laboratory tests [7]. Therefore, the present study aimed to determine the current rate of test repeats, their causes and usefulness, TAT, and costs associated with the repetition of biochemical laboratory tests before and after designing a protocol as a novel practice.

## 2. Materials and Methods

This cross-sectional study was conducted in the Clinical Chemistry Laboratory of Abu-Ali Sina Hospital (Shiraz, Iran), which is a liver transplantation center, from May 2020 to July 2020. This study was designed in accordance with the Declaration of Helsinki and carried out after obtaining approval from the Ethics Committee of Shiraz University of Medical Sciences (IR.SUMS.MED.REC.1399.020). Generally, the annual test volume for the clinical chemistry laboratory of the hospital is approximately seven million. To avoid false or erroneous routine results, repeat testing is performed for some critical and noncritical data, according to automated flags of autoanalyzers and also the operator's opinion, such as flags of linearity (values above AMR), delta checks, clinically significant values, and critical values. Although the cutoff values for AMR (linearity) and critical values are well known and documented, there are no strict cutoff points for clinical significance and delta checks; therefore, the repeat rules in our laboratory seem to be relatively subjective.

### 2.1. Study Design

To determine the current rate, causes, and usefulness of repeat testing, 26 tests were selected in the clinical chemistry laboratory: albumin, alkaline phosphatase (ALP), alanine aminotransferase (ALT), amylase, aspartate aminotransferase (AST), bilirubin total (BT), bilirubin direct (BD), blood urea nitrogen (BUN), calcium, chloride (Cl), total cholesterol (CholT), creatine kinase (CK), creatinine (Cr), glucose, gamma-glutamyl transferase (GGT), high-density lipoprotein-cholesterol (HDL-c), iron, lactate dehydrogenase (LDH), low-density lipoprotein-cholesterol (LDL-c), lipase, magnesium (Mg), phosphorus (Ph), total protein (ProtT), total iron binding capacity (TIBC), triglyceride (TG), and uric acid.  Table 1 shows the chemical methods, wavelengths, and the allowable CAP bias for all analytes [8].

All serum specimens from both hospitalized patients and outpatients, undergoing repeated tests over two consecutive months, were included in this study. Icteric samples were also included, as we could not avoid them in our center, which is a hepatic disease and transplantation center. The exclusion criteria were the specimens subjected to preanalysis errors, such as lipemia and hemolysis, and flags for values below AMR (mostly related to a low sample volume) that needed to be repeated with a new sample (the main sources of preanalysis error) [9, 10].

A laboratory investigation of the samples was carried out using two identical Dirui-1200 autoanalyzers. The results of the tests were obtained by running the separated sera after centrifugation. Two levels of internal quality control (QC) materials, that is, level 1 (normal) and level 2 (high), were evaluated in this study. Both analyzers were calibrated periodically and maintained daily for quality control. Also, the performance of the two analyzers was compared weekly, using regression analysis to confirm similar results. The repeat test runs were performed on the same sample in the same run on the same analyzer (as the initial one). Moreover, for the second month, we designed some test repeat rules, and all employees were required to follow them for repeat testing. The defined causes of test repeats in the protocol included:

(1) Linearity (high AMR): high cutoff values are extracted from the kit instructions.
(2) Delta check: it measures the difference between a patient's sequential test results [11]. The cutoff values are extracted according to the following formula:
  (1) $$\begin{array}{c} \text{RCV}=20\text{.}5\times 2\text{.}58\times \left(\text{CVA }2+\text{CVI }2\right)\text{0.}5\text{,} \end{array}$$
where RCV is the reference change value (allowable delta value); CVA is the analytical variation (from quality control); and CVI is the intraindividual coefficient of variation [8].
(3) Clinically significant values: the cutoff values are determined according to the medical references and also in coordination with the hospital clinicians [12].
(4) Critical values: the cutoff values are determined according to the references and in coordination with the hospital clinicians [2].

Table 2 presents our test repeat rules (cutoff ranges for defined causes). It should be noted that for some results, there were some overlaps between different causes.

### 2.2. Statistical Analysis

All data, including the dates, names of the tests, causes of test repeats, initial and verified results, time, and cost, for both consecutive months, were entered in Microsoft Excel for analysis. The absolute value and percentage of the difference between the two test runs were calculated for each value and then compared with the allowable bias limit. If the absolute difference between the final confirmed value and the initial result was greater than the College of American Pathologists/Clinical Laboratory Improvement Amendments (CAP/CLIA) allowable bias, the initial result was considered as an identified error, and the test was performed for the third time; the average of the two agreeing results was then reported. For comparison of data between the two consecutive months, Student's *t*-test was performed.

## 3. Results

A total of 142,480 biochemical tests and 7905 repeat tests were performed over two months, including routine (subjective) test repeats in the first month and rule-based repeats in the second month. A total of 191 test repeats were excluded from the analysis due to values below AMR, hemolysis, and lipemia (final sample volume: 7714). A significant decline (38%) in the repeated tests was found during the second month (*P* < 0.001) (Table 3).

**Figure 1:**
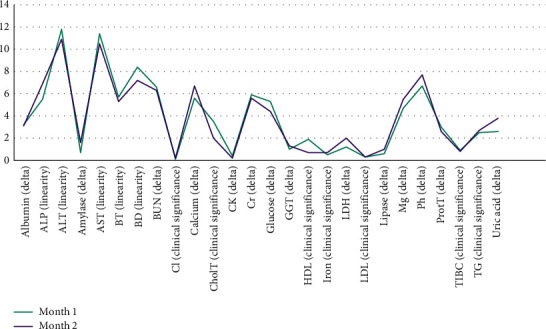
The percentage of each analyte in the total repeated tests and the most common cause of repeat for each analyte in two consecutive months.

**Figure 2:**
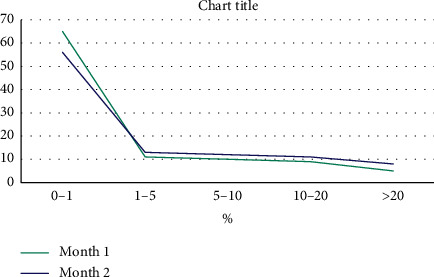
The bias pattern (mean) for all analytes over two consecutive months.

**Figure 3:**
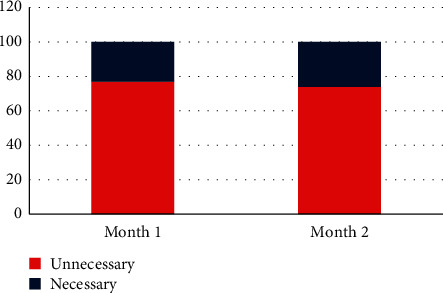
The percentage of unnecessary test repeats over two consecutive months.

The AST and ALT were the most commonly repeated tests in each month and also in both months.  Figure 1 shows the share of each analyte in the total repeat tests, as well as the most common cause of test repeats for each analyte in both months. The initial and repeated (verified) results were evaluated for various analytes, and the percentage of change in the values (bias) was calculated. There was no significant difference (<1% bias) between the initial and repeated (verified) results in 4666 (60.5%) repeated tests. As shown in Figure 2, the bias pattern was relatively similar in both groups, and comparison with the allowable inaccuracy level (bias) determined the percentage of unnecessary repeats in each month (Figure 3) [13].

**Figure 4:**
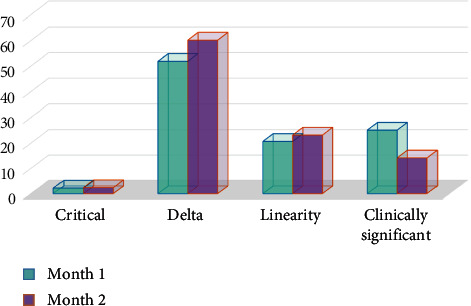
The distribution of different causes of repeat testing over two consecutive months in percentage.

Although in both groups, the most common causes of test repeats were delta values after protocol designation, there was a decline in the causes of clinically significant values, as shown in Figure 4. The Mg, ALP, AST, and lipase tests had the highest rates of necessary repeats, respectively (the least stable tests), while albumin, LDL, and CholT showed the highest rates of unnecessary repeats, respectively (the most stable tests). Besides, the total cost and delay in TAT due to repeat testing decreased by 32% and 36%, respectively.

## 4. Discussion

Although many strategies are used to eliminate laboratory errors, these errors still prevail [14, 15]. The improved reproducibility by intelligent automation has led to a reduction in the variability of results and errors of analysis, with remarkable improvements in the quality of laboratory results [2]. The practice of repeating a test to prevent false results is necessary when testing is performed using instruments with poor analytical precision [16]. In many laboratories, this practice has persisted for critical value measurements, despite major advances in areas, such as automation, precision, and quality assurance [16–18].

A summary of data from the College of American Pathologists (CAP) Q-Probes survey suggested that 61% of laboratories still repeated testing for critical values [19]. Generally, noncritical laboratory test results are not repeated before being available [6]. Elimination of routine repeat testing can help reduce the test cost and TAT. Our hospital is a referral inpatient and outpatient liver transplantation center with highly complicated cases and highly abnormal biochemical results. Therefore, we deal with a high number of repeated tests for confirmation in critical and noncritical ranges. To determine whether these duplicate tests can ensure or improve the accuracy of the results, we performed an audit of repeat testing to evaluate its usefulness, estimate the delay caused by repeat testing, and quantify the cost of repeating the tests.

In the present study, 7,714 repeat tests for 26 routine biochemical analytes were evaluated over two consecutive months to determine the accuracy of the results. In repeated test runs, 60.5% of the results showed no significant change after the test repeat (bias: 0–1%), and 75.5% of the repeats were within the acceptable limits of difference; therefore, they were considered unnecessary. Although there was a significant decline in the percentage of repeats, cost, and TAT in the second month with protocol designation, there was no significant change in the percentage of unnecessary repeats (77% in the first month vs. 74% in the second month). Also, delta check was the most common cause of test repeat in both months, although repeats due to clinical significance decreased after implementing the protocol, which could be due to the determination of objective criteria.

Overall, ALT and AST  were the most common repeated analytes in both evaluated months, each accounting for more than 10% of total repeats. This could be explained by the subspecialty of our center in hepatic diseases and liver transplantation, besides the high daily number of specimens with highly elevated liver enzymes that require at least one episode of repeat testing with dilution. The Mg, ALP, AST, and lipase tests had the highest number of necessary repeats, respectively (the least stable tests); the enzymatic nature of these analytes might make them so sensitive. On the other hand, albumin, LDL, and CholT were the tests with the highest unnecessary repeats, respectively (the most stable tests).

The number of studies evaluating the efficacy and necessity of repeat testing in clinical chemistry laboratories is limited, and most of them, including those by Niu et al. and Chima et al., have assessed a few analytes, only in critical ranges. However, these studies indicated the ineffectiveness of repeat testing and suggested its termination in clinical chemistry laboratories, except for values exceeding the AMR [16, 20]. In a similar study by Rodrigues et al., the usefulness of test repeat was evaluated for routine biochemical tests whether they were critical or noncritical. Overall, 81.3% of repeats were classified as unnecessary. Creatinine and urea were the most repeated tests, respectively, and among analytes that were common with our study, phosphorus, LDH, calcium, and ALP accounted for the highest percentages of necessary test repeats [21].

A comprehensive study by Deetz et al. evaluated the repeat laboratory results of 30 common laboratory tests (including 14 routine clinical chemistry analytes). In general, 3.8% of the total routine chemical analytes were repeated, which is consistent with our test repeat percentage after protocol designation. About 339 errors (necessary to repeat) were detected, most of which were related to values below AMR, followed by values above AMR and values within AMR (delta checks, critical values, and reviews). These findings suggest that when the initial results are within the AMR, repeated testing is unnecessary [7]. In another study by Onyenekwu et al., only 17 (0.7%) out of 2308 test repeats were significantly different from the initial values; however, only four analytes (sodium, potassium, calcium, and Mg) in the critical range were evaluated in this study [6].

Rule-based repeat testing in our clinical chemistry laboratory improved the number of repeated tests, costs, and TAT, without compromising the accuracy. Although the results of our investigation agree with those of previous studies, the necessity of repeat testing seems to depend on the laboratory type, and further evaluations in different types of medical centers are needed. For example, our center is a liver transplantation center with a high number of daily requests for liver function tests and many icteric samples, which potentially act as a source of error and interference in chemical tests. Therefore, dilutions must be used every day, even in the absence of high AMR (linearity) flags. Overall, each laboratory is suggested to follow its own policies and protocols for repeat testing and verification of the results. In this study, we assessed a high number of biochemical analytes in a highly variable and abnormal range. However, conducting experiments in a single specialized center, using only one type of automated analyzer, is still the main shortcoming of this study.

## 5. Conclusion

Although repeat testing has been shown to be unnecessary in most cases, having a strict policy for repeat testing appears to be more valuable than avoiding it completely. Each laboratory is advised to establish its own protocol for repeat testing based on its own practice.

## Figures and Tables

**Table 1:** Chemical methods, wavelengths, and the CAP allowable bias.

Analytes	Methods	Wavelengths (nm)	CAP allowable bias (%)	Reading time (min)
Albumin	BCG	546	1.43	10
Alkaline phosphatase (ALP)	DGKC	405	6.72	9
Alanine aminotransferase (ALT)	UV/IFCC	340	11.48	9
Amylase	Enzymatic	405	7.4	10
Aspartate aminotransferase (AST)	UV/IFCC	340	6.54	9
Bilirubin total (BT)	Photometric-2,4 dichloroaniline	546	8.95	10
Bilirubin direct (BD)	Photometric-2,4 dichloroaniline	546	14.2	10
Blood urea nitrogen (BUN)	Urease	340	5.57	4
Calcium (Ca)	CPC	620	0.82	10
Chloride (Cl)	Colorimetric	450	0.5	10
Cholesterol total (CholT)	Enzymatic	505	4.1	10
Creatine kinase (CK)	IFCC	340	11.5	10
Creatinine (Cr)	JAFFE	500	3.96	8
Glucose	GOD-PAP	500	2.34	10
Gamma-glutamyl transferase (GGT)	Enzymatic	405	11.6	10
High-density lipoprotein-cholesterol (HDL-c)	Enzymatic	600	6.9	10
Iron	Photometric test using ferene	600	8.8	15
Lactate dehydrogenase (LDH)	DGKC	340	4.3	9
Low-density lipoprotein-cholesterol (LDL-c)	Enzymatic	600	5.46	10
Lipase	Enzymatic	580	11.31	9
Magnesium (Mg)	Colorimetric	546	1.8	5
Phosphorus (ph)	Phosphomolybdate	340	3.38	6
Protein total (ProtT)	Biuret	546	1.36	10
Total iron binding capacity (TIBC)	Direct	660	1.3	10
Triglyceride (TG)	Enzymatic	505	9.57	10
Uric acid	Enzymatic	555	4.87	6

BCG: bromocresol green; IFCC: International Federation of Clinical Chemistry; DGKC: German Society of Clinical Chemistry; CPC: cresolphthalein complexone; GOD-PAP: glucose oxidase/peroxidase.

**Table 2:** The suggested protocol for repeat testing in our clinical chemistry laboratory.

Analyte	Unit	Reference range	Analytical measurement range (AMR)		Clinical significance		Delta check		Critical	
			Low	High	Low	High	Value	%	Low	High
Albumin	g/dL	3.5–5.5	0.2	6	3.5	5.7	0.5	10	—	—
ALP	IU/L	64–306	5	1000	60	380	40	20	—	—
ALT	IU/L	3–40	2	300	5	60	10	50	—	—
Amylase	IU/L	0–90	—	900	—	100	15	30	—	—
AST	IU/L	3–40	2	300	5	60	8	30	—	250
BT	mg/dL	0.1–1.3	0.1	30	—	1.4	0.2	20	—	18
BD	mg/dL	0.1–1.3	0.1	10	—	0.3	0.2	100	—	—
BUN	mg/dL	6–20	4.4	140	5	20	5	30	—	80
Calcium	mg/dL	8.5–10.5	5	15	7	10.6	1	10	6	13
Cl	mmol/L	98–110	25	300	90	112	5	5	80	120
CholT	mg/dL	100–200	5	500	—	200	30	20	—	—
CK	IU/L	0–171	10	1700	—	240	60	70	—	—
Cr	mg/dL	0.6–1.4	0.2	20	—	1.5	0.2	20	—	5
Glucose	mg/dL	70–100	20	400	55	126	20	20	40	400
GGT	IU/L	0–49	—	284	—	60	10	40	—	—
HDL	mg/dL	40–60	5	100	40	60	10	20	—	—
Iron	*µ*g/dL	40–165	5	500	40	220	70	80	—	—
LDH	IU/L	235–470	50	1200	—	470	100	25	—	—
LDL	mg/dL	60–130	1	400	—	100	15	25	—	—
Lipase	IU/L	0–60	5	250	—	60	40	60	—	—
Mg	mg/dL	1.5–2.5	0.5	5	1.5	2.5	0.2	20	1	4.7
Ph	mg/dL	2.7–4.5	2	20	2.7	4.5	0.5	20	1	8.9
ProtT	g/dL	6.6–8.8	0.5	15	5	8.8	1	10	—	—
TIBC	*µ*g/dL	250–450	77	694	220	450	30	10	—	—
TG	mg/dL	40–150	5	700	40	150	50	60	—	—
Uric acid	mg/dL	2.6–7.2	0.5	25	2	8	1.5	30	—	13

**Table 3:** Total tests and repeat tests over two consecutive months.

Month	Total tests	Repeat tests (%)
1	69,430	4656 (6.7)
2	73,050	3058 (4.1)
Total	142,480	7714 (5.4)

## Data Availability

The data used to support the findings of this study are included within the article.
